# Biocompatible chitosan–collagen–hydroxyapatite nanofibers coated with platelet-rich plasma for regenerative engineering of the rotator cuff of the shoulder

**DOI:** 10.1039/c9ra03972d

**Published:** 2019-08-28

**Authors:** Yi Tang, Hui Zhang, Qinghua Wei, Xu Tang, Wanqiang Zhuang

**Affiliations:** Department of Orthopedics, People's Hospital of JianYang Sichuan Province China hui.zhang26@yahoo.com

## Abstract

Over the last few decades, extraordinary progress has been accomplished in the field of bone tissue engineering. Containing an incredible number of growth factors required for the process of osteogenesis, platelet-rich plasma (PRP) has gained much interest. However, because of the conflicting results obtained in various investigations, its adequacy remains a riddle. Accordingly, in this paper, we explore the *in vitro* application of biocompatible chitosan–collagen–hydroxyapatite (CS–COLL–HAP) nanofibers coated with platelet-rich plasma (PRP) (CS–COLL–HAP/PRP) scaffolds for the regenerative engineering of the rotator cuff (RCF) of the shoulder. FTIR spectroscopy, XRD, SEM-EDX and HRTEM were performed to evaluate the characteristics of nanofibers. After confirmation of the physicochemical properties of nanofibers, the osteogenic capability of the scaffold was assessed by measuring the relative calcium content, ALP activity, and gene expression. The results of viability and live/dead assay and cell adhesion test indicated the adequacy of the PRP when coupled with nanofibers in contrast to the other tested groups. *In vivo* staining affirmed increased collagen association in the PRP with nanofiber scaffolds at 30 days and 60 days. In conclusion, the addition of the PRP into CS–COLL–HAP nanofibers in this examination affected the osteogenic differentiation of osteoblast cells, and therefore, it may have an incredible perspective for bone tissue applications.

## Abstract 2

Over the last few decades, extraordinary progress has been accomplished in the field of bone tissue engineering.

## Graphical abstract

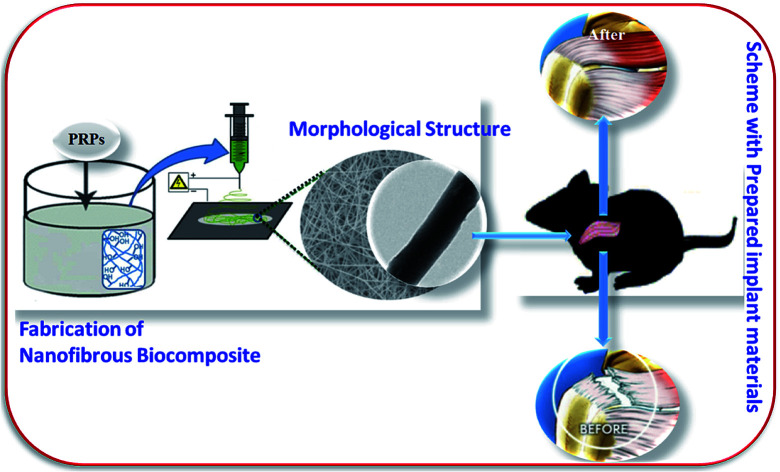

## 1. Introduction

In 2011, the treatment of musculoskeletal infections, disorders, and injuries costed more than 175 billion dollars.^1^ Shoulder-related disorders influence critical segments of the populace, and particularly, the old, with symptoms being crippling pain, diminished capacity, and joint precariousness.^2^ In view of 2010 studies, around 5 million individuals in the USA have some dimension of discomfort in the shoulder, requiring doctor's visits for more assessment.^3^

Of the 5 million appointments, roughly 4 lakh patients underwent surgeries to treat rotator cuff injuries.^4^ The problems of high expensive surgeries and United follow up nonoperative medicines, high disappointment rates running between 20 and 70% have been accounted for after RCF surgical repair.^5^ High disappointment rates are ascribed to firmness, disease, fatty invasion, muscle decay, muscle withdrawal, and RCF degeneration.^5^ An ongoing examination has proposed that the re-tear rates are profoundly subject to the preoperative tear measure.^6^

There has been increasing interest for optional or accessorial medications in bone therapy using PRP to help in healing.^7–9^ Administration of PRP to the surgical site has been utilized as an approach to profit by the hemostatic role that platelets play in cluster development, development factor discharge, and recuperating.^10,11^ A lot of *in vitro* work has been done to describe PRP and the mechanism underlying platelet initiation and ligament cartilage recuperation resulting in extraordinary achievements.^12,13^ This creates the absence of medical efficacy all the more confounding and has restricted broad utilization of PRP execution in bone tissue methodology.^14,15^ The lack of clearness in biomedical strategies might be ascribed to the administration techniques and the lack of supported conveyance cargo. With present administration strategies, there is little difficulty in delivering the PRP to the target site. Therefore, it is perfect to have a PRP carrier to give supported discharge all through the recuperating procedure.^16^

In this investigation, bioactive CS–COLL–HAP nanofibers with and without PRP were prepared using the electrospinning technique. The obtained nanofibers were characterized using FT-IR spectroscopy, XRD and SEM techniques. Nanofiber implants were tried in ovine meniscus repair models, where they improved cell recruitment, rebuilding, vascularization and the renovation of tissue integration in comparison with the addition of PRP or wrapping of the meniscus with a COLL layer.^30,31^ In conclusion, the prepared nanofiber inserts improved marrow-animated ligament repair and initiated bone remodeling in the animal model. We theorized that the majority of the above techniques would likewise be beneficial for RCF, and we subsequently demonstrated in a rat model that the prepared nanofiber implants improve trans-osseous RCF *via* favoring ligament connection by expanded bone tissue remodeling.

## 2. Materials and methods

### 2.1. Fabrication of nanofibers

The acquired CS–COLL scaffold together with pure CS, COLL, and HAP was then used to formulate a solution for electrospinning the nanofibers of CS–COLL–HAP (NF2), with CS, COLL, and HAP stacked at weight proportions of 28.9 wt%, 3.6 wt%, and 13.9 wt%, respectively. An aqueous solvent mixture containing 3 wt% acetic acid and DMSO in a weight ratio of 10 : 1 was used to dissolve the compounds at RT. After being vigorously mixed, the solution was drawn into a syringe with a tip needle appended. The syringe was mounted onto a syringe pump, and the needle was connected to a high-voltage control. On applying 15 kV voltage, the liquid discharged and the resultant nanofibers were collected in an aluminum foil. The CS–COLL (NF1) scaffold was prepared additionally and taken as a control sample.

### 2.2. Characterization of samples

The chemical structure of the prepared scaffold was investigated by FTIR spectroscopy using a Thermo Scientific spectrometer. The crystalline phase of the prepared scaffold was investigated by XRD using Cu Kα radiation, in the range of 10–70°. The microscopic images of the prepared nano-fibers were obtained using a SEM. The HRTEM images of the prepared nano-fibers were obtained using a transmission electron microscopic instrument.

### 2.3. Viability and live/dead cell assay

Cell multiplication and morphologies in prepared samples were examined by MTT assay and fluorescein diacetate/propidium iodide recoloring. The foreordained time points for the identification were 1, 7, and 2 days. In the MTT test, osteoblast cell-loaded samples were first drenched in MTT at RT. After half-a-day brooding, the solution was expelled and DMSO was, in this manner, added to break up the purple MTT salts. Ultimately, a microplate reader was used to quantify the absorbance of the arrangement. Each of them was repeated thrice. For cell compatibility analysis, the nano-fibers were immersed in fluorescein diacetate/propidium iodide solution and imaged using a confocal laser examining magnifying lens.

### 2.4. Histological analysis

All experiments were performed in compliance with the animal protection law of the People's Republic of China following the IACUC guidelines. A cut was made over the correct shoulder in female SD rodents weighing 200–250 g. The tendon was isolated, and the deltoid was along these lines to imagine the supraspinatus ligament. A nylon suture was used to verify the ligaments utilizing a modified M-A suture. Once verified, the ligament was totally dismembered from the humeral head. A needle was utilized to cut a gap through the humeral head and the stitch was strung through the subsequent opening. At the implantation site, the rodents either got no extra careful control, an embedded CS–COLL–HAP and CS–COLL–HAP/PRP nanofibers. The ligament was then verified deep down, the deltoid sewed together, and the entry point site shut. The test samples were reaped at 1 month and 2 month time intervals. They were rinsed with phosphate buffer solution for 20 min each, washed with ethanol for 20 min each and then dried before embedding in paraffin wax. The samples were segmented and stained with H&E, picrosirius red and trichrome.

## 3. Results and discussions

### 3.1. Spectral characterization

**Fig. 1:**
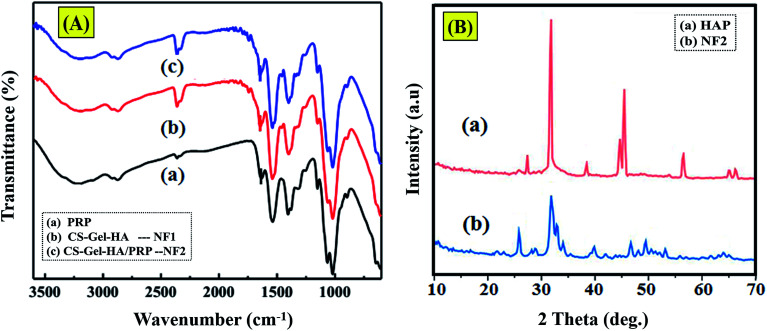
(A) FTIR spectrum of prepared samples. (B) XRD images of prepared samples.

The FTIR spectrum of the platelet-rich plasma displayed amide I and II bands at 1555 cm^−1^ and 1665 cm^−1^, individually, and a wide band in the 3335 cm^−1^ area assigned to N–H bunches from the platelet-rich plasma (protein).^17^ Looking at the spectra in Fig. 1A(a) and (c), the characteristic amide II band was evidently moved to 1580 cm^−1^ in the NF2 scaffold. In the meantime, its intensity is superior to that of the amide I peak in the NF2 scaffold. In Fig. 1A(b), the peaks at 569, 605 and 1033 cm^−1^ are attributed to the characteristic vibrations of P–O gatherings of apatite.^18,19^ Interestingly, the non-covalent bonding of PRP led to the shift in the peak position of the C=O component (Fig. 1A(b)).^15,17^

XRD was used to recognize the diffraction planes of the prepared samples, HA and NF2 scaffold. We used commercial hydroxyapatite as control and demonstrated that the formation is kind of practically equivalent to HAP (Fig. 1B). Thereinto, the characteristic 2*θ* planes of hydroxyapatite were seen at angles of 31° and 27°.^20^ They were not as sharp as the portrayed diffraction planes of commercial HAP, which outlined that the hydroxyapatite was of only a moderate level of crystallization.^21,22^

### 3.2. Morphology characterization

**Fig. 2:**
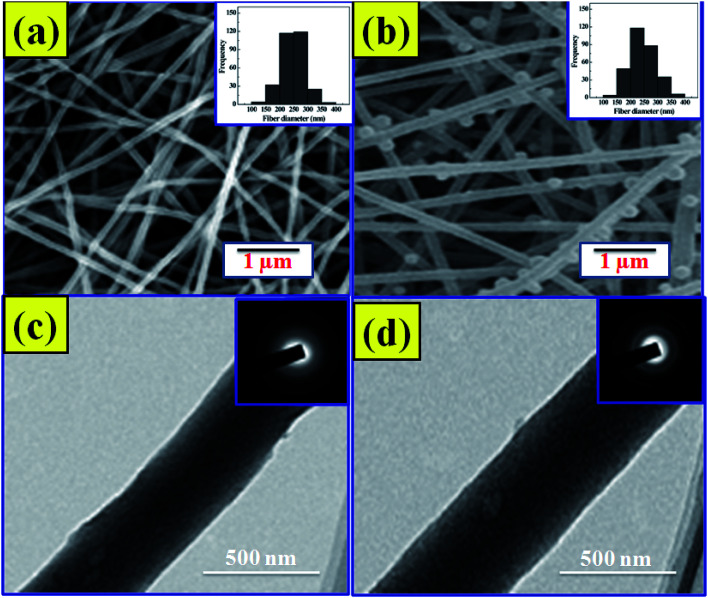
SEM images of NF1 (a) and NF2 (b). HRTEM images of NF1 (c) and NF2 (d).

The SEM images and the comparative width distributions are shown in Fig. 2a and b, respectively. The structure of the nano-fibers is observed to be flat and homogeneous.^23^ The nanofibers are randomly arranged with high porosity. The average width of the NF1 scaffold was observed to be 250 ± 105 nm. The PRP incorporation into NF2 scaffold builds the normal distance across increments from 250 ± 105 nm to 280 ± 146 nm. With the addition of PRP into the CS–COLL–HAP lattice, the breadth of the nano-fibers expanded.^24^ The morphology of the prepared samples was additionally described by utilizing HRTEM. Despite the fact that NF1 demonstrates a huge differentiation in the brilliance at the center and shell (Fig. 2c), any recognizable distinction in the brightness is not observed for NF2 (Fig. 2d).

### 3.3. Viability and live/dead cell assay

**Fig. 3:**
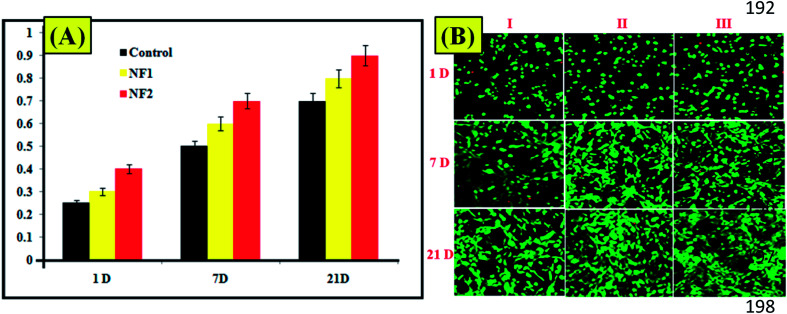
(A) Viability assay of prepared nanofibers. (B) CLSM (live/dead cells) images of (I) control, (II) NF1, and (III) NF2.

The live/dead status, proliferation, and adhesion of osteoblast cell in prepared specimens are displayed in Fig. 3B. As can be seen from the confocal laser scanning microscopy images,^25^ osteoblast cells in NF1 and NF2 scaffolds showed high viability. With respect to osteoblast cells, NF2 nano-fiber scaffolds demonstrated an essentially higher proliferation rate than the others on day 7, while NF1 demonstrated a slower increment and a lower cell thickness (Fig. 3A and B). The images (Fig. 3B) show that osteoblast cells in each of the three groups started to frame extensions, despite the fact that a portion of the osteoblast cell still stayed around after 1 day incubation. The number of spread cells and their spreading augmentation expanded with the brooding time. Eventually, osteoblast cells in the NF1 and NF2 nanofiber scaffold formed into a regular axle figure, in which the outgrowth was evident.^26^ However, most cells in the control retained the round or controlled shape.

### 3.4. Cell adhesion

**Fig. 4:**
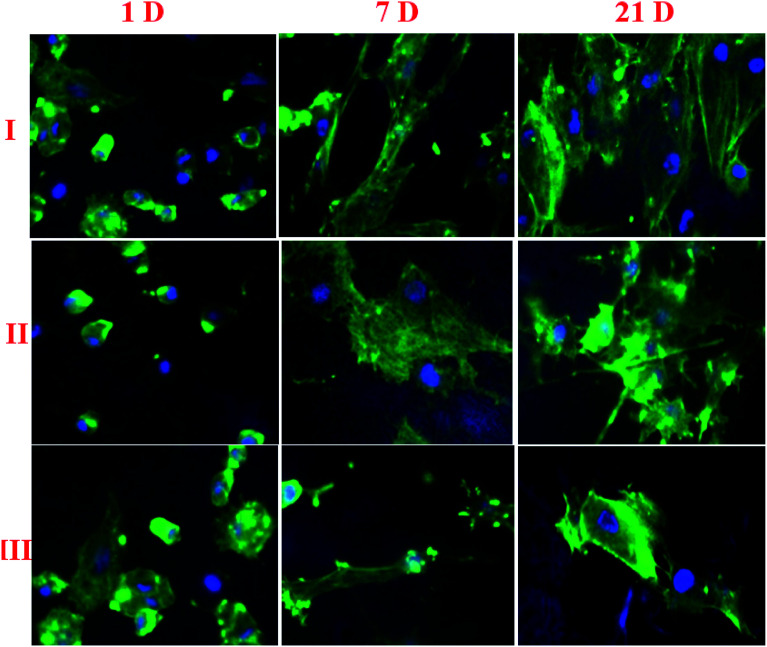
Osteoblast cell adhesion on (I) control, (II) NF1, and (III) NF2 samples after different days of cultivation.

The cell adhesion structure and development of osteoblast cells on NF1 and NF2 nano-fibers were pictured using CLSM. The osteoblast cell attached and expected particular structures on the two sorts of nano-fiber was exhibited in Fig. 4. In particular, the rotator cuff fibroblast cells developed on the NF2 filaments exhibited a phenotypic prolonged morphology and was arranged along the nanofiber long axis. Interestingly, rotator cuff fibroblast cells seeded on the NF2 nanofiber scaffold showed a polygonal structure without special direction. In addition, as rotator cuff fibroblast cells multiplied on the two sorts of nano-fibers, these structure contrasts were kept up over the 21 day incubation period (Fig. 4).

### 3.5. Osteogenic differentiation

**Fig. 5:**
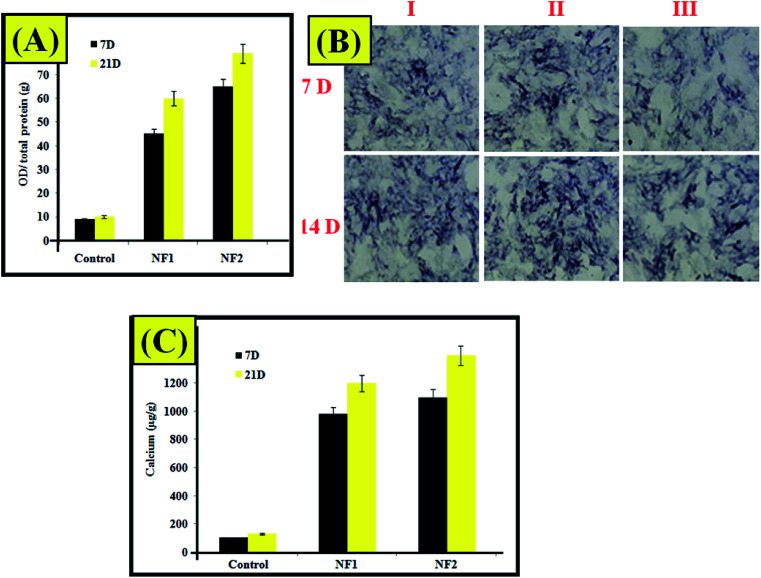
(A) ALP assay. (B) Alizarin red staining (I) control, (II) NF1, and (III) NF2 samples. (C) Calcium content assay.

As shown in Fig. 5A, on day 7, the NF2 scaffold demonstrated the most increased amount of alkaline phosphatase. The greater amount of alkaline phosphatase on day 14 contrasted and the control gathering had a place with NF1, which were individually improved. On day 14, the calcium content of NF2 analyzed in the control group expanded, which was the best in contrast to different groups. Considering the outcomes from alkaline phosphatase movement and calcium content tests, one can arrive at the resolution that the platelet-rich plasma-incorporated nano-fibers demonstrated the best outcomes.^27,28^Fig. 5B demonstrates AR stains of the prepared samples. Osteoblast cell connection on the nanofiber and low cell in growth were occurred as its daily practice for scaffold.^29^ In addition, the NF2 makes more mineralization when compared with different groups. Despite the fact that the aftereffects of staining are not solid enough all alone, they are in accordance with the ones from alkaline phosphatase movement and calcium content tests (Fig. 5C).^29^

### 3.6. Gene expression

**Fig. 6:**
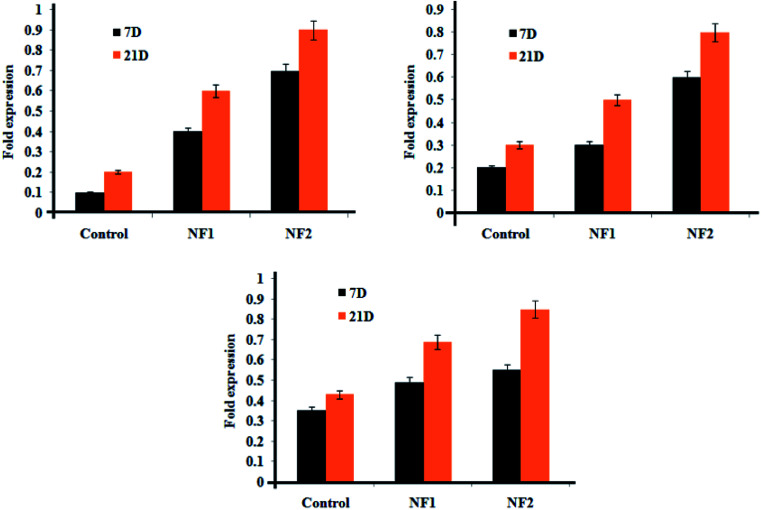
Gene expressions of osteoblast cells after 7 and 21 days of incubation.

Gene expressions of cell-related separation in the osteoblast-like gathering were recognized and evaluated by qRT-PCR (Fig. 6). Osteoblast cell-loaded HAP was utilized as the control group. In gene choice, COLL-I, OCN, and ALP were picked as markers for osteogenesis separation of osteoblast cells. In all groups, all gene expressions considerably expanded with delayed culture time (from 7 to 21 days). There was no noteworthy distinction between the NF1 gathering and the mass NF2 for OCN and ALP expressions, while the COLL-I expressions were clearly improved in the get together in the meantime point.

### 3.7. *In vivo* rotator cuff fibroblast cell regeneration

**Fig. 7:**
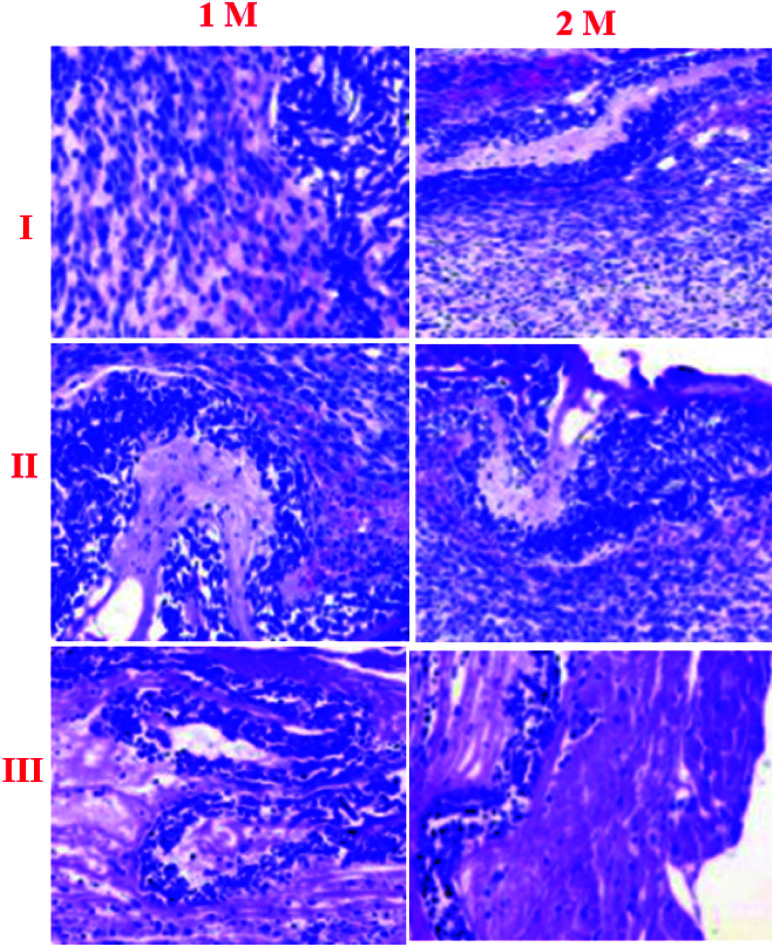
Bone remodeling, as confirmed by H&E staining (I) control, (II) NF1, and (III) NF3 samples.

Haemotoxylin and eosin staining were performed for qualitative surface appraisal of the repair.^30^ Both repair types showed indications of recovery as shown by vascularization and expanded cell thickness. However, there were particular contrasts. Haemotoxylin and eosin staining demonstrated that the NF2 repair was commonly made out of evaluated regions similar to local enthesis morphology (Fig. 7). This region arrangement was not seen in the negative repairs, which had less fibro-cartilage joining and less composed COLL strands than NF2. In general, control repaired shoulders were described by an unexpected limit of blemish-like tissues at the ligament cartilage interface (Fig. 7).

**Fig. 8:**
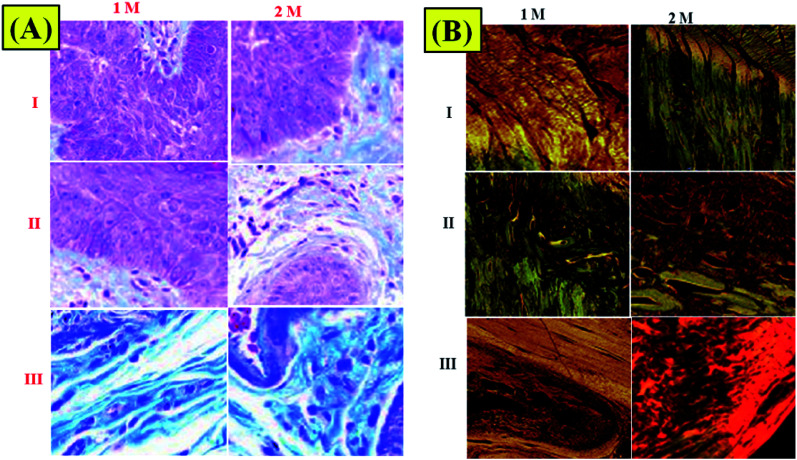
(A) Trichrome staining of surgically repaired rat rotator cuffs. (B) Picrosirius red-stained histological images of (I) control, (II) NF1, and (III) NF2 samples.

Masson's trichrome is useful for recognizing COLL from skeletal tissue encompassing the shoulder joint.^31^ The nearness and association of COLL can be effectively pictured using this kind of Masson's trichrome stain (Fig. 8A). Disorderly COLL and higher cell densities as depicted above can be seen at 1 M. At 2 M, more COLL was available close to the interface in the NF1 and NF2 nano-fiber scaffolds. The NF2 nano-fiber scaffold is the main gathering that displays higher degrees of COLL pack association at the interface like the control muscle. This appearance aids in the increment of mechanical quality for NF2 nanofiber scaffolds at 2 M. The groups at 84 d are in the redesigning stage with low cell densities and more collagen at the ligament bone interface. The control and NF2 specimens at 2 M have a recognizable reintegration of the ligament into the cartilage that is further developed compared with different groups at 2 M.

Picrosirius red staining was used to assess tissue COLL arrangement at the enthesis border.^32^ The results of COLL arrangements demonstrated that NF1- and NF2-implanted shoulders had fundamentally more noteworthy COLL arrangement contrasted with control specimen implanted shoulders (Fig. 8B). The expansion force of NF2 fixes recommends an increment in renovating of the COLL design to an appropriately sorted out COLL system and looks like local enthesis. Conversely, the control specimen seems, by all accounts, to contain complicated disfigurement tissue at the interface.

## 4. Conclusion

This investigation focused on the design, characterization, and methodical *in vitro* and *in vivo* assessment of a novel bio-mimetic, nano-fiber (CS–COLL–HAP/PRP)-based framework for shoulder joint repair. It has been discovered that CS–COLL–HAP/PRP scaffold association significantly affects the osteoblast reaction, with the basic anisotropy of the adjusted platform straightforwardly directing cell adhesion, gene articulation, and biomineralization. Restricted cell reaction resulted in a bio-mimetic framework for the shoulder joint on the adjusted nano-fiber platform, and the biologically applicable platform was retained *in vitro* and *in vivo*. The findings of this examination reveal that the novel nano-fiber (CS–COLL–HAP/PRP) framework has huge potential for ligament recovery and leads to a utilitarian tissue-designing for shoulder joint repair. Future work will include a large number of animal models with further development, having the long haul target of applying this innovation to the clinic.

## Conflicts of interest

There are no conflicts to declare.

## Supplementary Material
